# The Integration of Global Competence Into Malaysian English as a Second Language Lessons for Quality Education (Fourth United Nations Sustainable Development Goal)

**DOI:** 10.3389/fpsyg.2022.848417

**Published:** 2022-06-20

**Authors:** Nur Syafiqah Yaccob, Melor Md Yunus, Harwati Hashim

**Affiliations:** ^1^Sekolah Menengah Kebangsaan Dato’ Abdul Rahman Ya’kub, Melaka, Malaysia; ^2^Faculty of Education, Universiti Kebangsaan Malaysia, Bangi, Malaysia

**Keywords:** ESL, global competence, SDG 4, teaching and learning of English, quality education

## Abstract

Research in the globalization era has emphasized the importance of global competence and its integration into language teaching and learning. This article discussed the current focus of global education through the integration of global competence in English as a Second Language (ESL) in Malaysia. The country’s global competence development among ESL teachers and students, along with its integration into English lessons, were identified, satisfying the need to achieve quality education. The global competence integration of ESL teachers will meet the fourth United Nations Sustainable Development Goal (SDG 4), which strives for quality education. The global education goals form a critical starting point of this article, which articulates the connection to the targets in SDG 4. Hence, this article investigated the significance of global competence in language teaching and learning in Malaysia. Overall, the integration of global competence among ESL teachers can provide life-long learning, enhancing the quality of education.

## Introduction

The recent years have seen language skills in English teaching dominating the edification of content. Historically, the teaching and learning of English as a second or foreign language has been emphasized as the domain of teaching language skills worldwide. These skills are necessary for picking up a new language, encompassing grammar, reading, writing, listening, and speaking. Scholars perceive that English lessons should not be limited to vocabulary, grammar, and skills but the language background and worldwide content (Hesan et al., 2019; Djumanova, 2020). Incidentally, globalization has widened the scope of language education to achieve global education goals.

The goals (1) prepare the future generations to live, work, and cooperate with diverse others. Furthermore, (2) a creative and reflective personality can be developed, enhancing the individuals’ constructive decisions and responsibility. Additionally, (3) the students’ global competence can be enhanced to productively function in their community and global society (Sinagatullin, 2019). In the long run, these goals contribute to learning by enhancing global competence among ESL teachers and students (Boeren, 2019; Mansilla and Wilson, 2020). Students can use the target language to deliver appropriate content for authentic interaction, consequently developing a global self-identity. This phenomenon is attainable when students can learn to think and communicate through an intuitive understanding of language (Djumanova, 2020).

In achieving the global education goals, the United Nations launched the Sustainable Development Goals (SDGs) in 2015 with “17 goals to transform the world” (Amponsah et al., 2018; United Nations, 2020). Through the Global Goals, the United Nations (2020) aimed to emphasize and generate actions from all countries to promote economic growth and social needs, i.e., education. The Global Goals is designed to help ESL teachers to shoulder the role of social workers, working toward social change and advocacy for human rights using English as a medium (Ait-Bouzid, 2020; Williams, 2020). In Malaysia, ESL teachers are considered bridge-builders between society and individuals through ESL teaching (Boeren, 2019; Nakidien et al., 2021). The diverse roles played by these teachers are examples of measures that ultimately empower global education in the country’s curriculum.

The existing literature highlights the significance of global competence integration in achieving the SDGs (OECD/Asia Society, 2018), which centers on transforming education. The reformation idea must align with the current and future economy, environment, health, and human needs (Boeren, 2019). By 2030, this integration is anticipated to address, manage, and solve the SDGs’ social, political, economic, and environmental issues with the support of teachers and students (OECD/Asia Society, 2018). Notably, various SDG targets are easy to achieve with impactful and accessible education and training systems (Boeren, 2019). Training includes teacher education and professional development programs for English teachers. The SDGs aim to provide quality global education by increasing access to education and achieving minimum proficiency standards. Others include assisting countries in connecting and facilitating inclusive learning opportunities. Hence, Malaysia’s Ministry of Education (MoE) must act swiftly to accomplish this idea through the SDGs in our education system (Zulkifli and Ahmad, 2021).

Given the points above, this paper aims to determine the relevance of global competence in ESL classrooms. This idea is grounded on current critical points vis-à-vis Malaysian ESL teachers’ global competence in achieving the SDG for quality education. Furthermore, this paper asserts that lifelong learning for students upon integrating global competence among ESL teachers in English lessons.

### Global Competence for Sustainable Development Goal 4: Quality Education

Global competence in education helps educators, teachers, and students comprehend local, global, and multicultural content. OECD/Asia Society (2018) defines the domains of global competence as the investigation of the world, recognizing perspectives, communicating ideas, and taking actions (OECD/Asia Society, 2018). Alternatively, this term can be defined as the ability to examine local, international, and intercultural issues (Tichnor-Wagner et al., 2016; OECD/Asia Society, 2018). Collective well-being and sustainable development entail the critical understanding and acceptance of other perspectives. Additionally, it is crucial to implement an effective relationship with individuals from diverse cultures.

Moreover, the global competence model presents the eight domains of global competence categorized by several constructs (Hunter, 2004). These constructs include (a) ability to know oneself (self-awareness), (b) approach and relationship with others, (c) knowledge of significant cultures worldwide, and (d) interpersonal skills (Global Competence Associates, 2021). However, various elements in Hunter’s (2004) global competence model overlap with several SDG global goals students are exposed to at school. These elements comprise the community of intercultural awareness, knowledge of global events, and valuing diversity. The goals include learning the well-being, gender equality, water, and climate change in science and language subjects (Williams, 2020).

The significant integration of critical global literacies into the lesson has been articulated with real-world issues and students’ global engagement (Yoon et al., 2018). These global topics and issues are discussed in ESL lessons, enlightening the students regarding the worldwide phenomenon. Other SDGs include increasing employment, eliminating gender disparities, and acquiring literacy, numeracy, knowledge, and skills to promote sustainability through education (United Nations, 2020). Similarly, global competence in the OECD PISA framework combines knowledge, skills, attitudes, and values (Hunter, 2004; OECD, 2018). The elements and goals are related from the perspectives of achieving comprehensive education through developing individuals’ global competence in skills and morality.

The critical SDG 4 necessitates all youth, regardless of gender, to achieve literacy by 2030. However, it was reported that 617 million youth worldwide are either illiterate or lack fundamental reading abilities. Half of the global illiterate population is located in South Asia, and a quarter is in sub-Saharan Africa (United Nations, 2020). Students face challenges understanding foreign topics and issues presented in textbooks, while others struggle to relate to the context. Thus, this predicament affects English language acquisition, especially when it is not grasped in an appropriate and authentic context. Moreover, a long-standing line of critique highlighted the government’s inefficiency in integrating information into their national English curriculum, connecting the country’s societal context (Amponsah et al., 2018).

In Malaysia, cultural content is biased in the Common European Framework of Reference (CEFR)-aligned English curriculum textbooks. Despite its holistic and deep language learning approach for ESL students, this predicament still prevails. The CEFR essentially describes the requirements for language learners, specifically as social agents, to achieve interaction and communication in English (Cagatay and Gurocak, 2016; Uri and Aziz, 2018). However, the textbook content is limited to articles and examples primarily from the “American, ““British,” or “European” cultures. As a result, the content overlooked other cultures globally, such as Asian and Middle Eastern nations (Galante, 2015; Johar and Abdul, 2019).

Individuals should be able to learn English authentically and grasp the ability to relate to their surroundings as language and cultures are interrelated (Council of Europe, 2020). They can achieve global competence by being cognizant of the global issues, knowledge, and cultures that enrich their local identities. However, a Malaysian textbook analysis by Johar and Abdul (2019) reported minimal to zero concerning the country’s cultural context. The Malaysian ESL teachers reported that the Pulse 2 textbook content has limited relevance to their students based on their respondents. Accordingly, the teachers find it challenging to comprehensively engage with the various topics and content. Similarly, the lack of indigenous voices in the English syllabus should be noted (Hauerwas et al., 2021). A more inclusive approach to teaching must be encouraged that includes indigenous people as part of crucial global awareness (Hauerwas et al., 2021). Given these points, the global competence of English teachers potentially aids the limited global and local perspectives found in the local textbooks.

ESL teachers should situate their teaching where their students are, locally and developmentally (Hauerwas et al., 2021). This idea creates inquiry experiences, enabling students to discover local and personal connections while broadening their perspectives. Furthermore, ESL lessons must be incorporated with universal issues as English is a global language, connecting various ideas (Yaccob et al., 2021). Thus, this approach mitigates illiteracy and reduces ill-informed students on such topics (Yoon et al., 2018) such as human rights and gender equality. Other critical matters include promoting a culture of peace and non-violence, global citizenship, and appreciating cultural diversity and contribution (United Nations, 2020).

In essence, students will grasp and appreciate knowledge if they experience it through learning, contributing to their global well-being (Bakar et al., 2021). Several approaches are suggested for global competence, i.e., structured debates on global issues, current events discussion, organized discussions to express perspectives, and flexibility to new information (OECD/Asia Society, 2018). Others include collaborative activities, project-based learning for authentic projects, conflict management, and activities for the ESL students to deepen their knowledge. Reimagining the language classroom as a diverse global community potentially defines its significance in the local community.

### Malaysian ESL Teachers’ Global Competence in English Classrooms

The global education and SDGs aim to substantially increase youth and adults with relevant skills in the real world (Amponsah et al., 2018). For instance, Williams’ (2020) ongoing Goals Project assists students in comprehending global challenges, constructing knowledge, and generating solutions. However, this project is not specific to the English lessons but encompasses all subjects. It would, therefore, be beneficial if a similar Goals Project that focuses on ESL lessons could be organized, catering to specific groups of ESL teachers and students. In Malaysia, various challenges emerge in providing the requirements for professional development programs for ESL teachers. These challenges comprise implementing global competence in ESL lessons, including insufficient awareness, support, and resources from the MoE (Sukri et al., 2017).

Significant investments must be made in developing professional development programs for ESL teachers to ensure global competence, which is the core of educational practice (Sukri et al., 2017; OECD/Asia Society, 2018). Global competence development does not require massive funds or extraordinary English teachers (OECD/Asia Society, 2018). However, these programs should not be limited to improving their linguistics proficiency but to include the pedagogy to develop global content. The ESL teachers are the mediator connecting international and local issues, thereby clarifying the local perspectives (Hauerwas et al., 2021). This idea is fundamental, especially in teaching and working in Malaysia’s diverse communities (Yaccob et al., 2021). Ultimately, lifelong learning can be initiated where students learn to communicate effectively and responsibly, establishing shared understandings and solving problems (OECD/Asia Society, 2018).

Technological advancement has made global connections simpler and feasible, expanding the world’s connectivity (OECD/Asia Society, 2018). Hence, there is a pressing need for students to be globally competent. Students must be educated and extend their learning for employability and livelihood to achieve the goals of quality education in SDG 4 (OECD/Asia Society, 2018; Williams, 2020). In the present climate, it is critical to establish language classrooms that identify students’ needs to work in a complex environment with people from diverse backgrounds. Incorporating global competence for ESL teachers in lessons provides meaningful interaction and collaboration in international and future work environments. The integration enables students to prepare for complex global societies by understanding the global scenery, ultimately improving their well-being (Mansilla and Wilson, 2020). This idea extends the ESL teaching and orientation toward the students’ future needs in a timely approach.

## Discussion

Considerable gaps continuously exist related to educational provisions in the academic system of various countries. SDG 4, therefore, aims to reduce these gaps by implementing and maintaining high-quality education systems (Boeren, 2019). Malaysia’s ESL teachers must determine the method in attaining SDG’s inclusive and accessible quality education. Accordingly, this approach should be implemented through language teaching to attain in the nation (Boeren, 2019; Nakidien et al., 2021). As a potential future development in the field, the MoE must organize a systematic program to develop the global competence of English language teachers. This effort can be supplemented with critical support from educators and researchers worldwide, committing to global collaboration. Global collaboration can be conducted to diversify research on global competence and quality education, which is not limited to the western perspectives. This idea is specific to the cultural and ideological work in non-native English-speaking countries such as Malaysia.

In essence, the proposed interpretation of global competence for ESL teachers and quality education will help the teachers in and beyond Malaysian schools. The development may support incorporating relevant international and local context practices in ESL lessons. Notably, the world is becoming increasingly culturally diverse and economically interdependent. Thus, various educational endeavors should initiate the development of globally quality students and future employees.

## Conclusion

Numerous ESL teachers are not aware of the elements of global competence and how they can be developed and integrated into English lessons. Furthermore, the lack of research on integrating global competence into ESL lessons in Malaysia is disheartening when it should have been emphasized to complement the current CEFR-aligned curriculum. Hence, this mini-review contributed to the literature on global competence in the ESL context, serving as a reference for future research in a similar area. Additionally, the outcome of this paper could inform educators and researchers about the current education reform and global competence in education, specifically for the English language.

## Author Contributions

All authors listed have made a substantial, direct, and intellectual contribution to the work and approved it for publication.

## Funding

This manuscript is funded by a grant from the National University of Malaysia and Universiti Kebangsaan Malaysia funded this research with grant no. GG-2020-024.

## Conflict of Interest

The authors declare that the research was conducted in the absence of any commercial or financial relationships that could be construed as a potential conflict of interest.

## Publisher’s Note

All claims expressed in this article are solely those of the authors and do not necessarily represent those of their affiliated organizations, or those of the publisher, the editors and the reviewers. Any product that may be evaluated in this article, or claim that may be made by its manufacturer, is not guaranteed or endorsed by the publisher.
